# Supplementary figures and images for: Reduced CXCL1 production by endogenous IL-37 expressing dendritic cells does not affect T cell activation
Source: PLoS One. 2021 May 24;16(5):e0251809. doi: 10.1371/journal.pone.0251809 (PMC8143410; doi:10.1371/journal.pone.0251809)

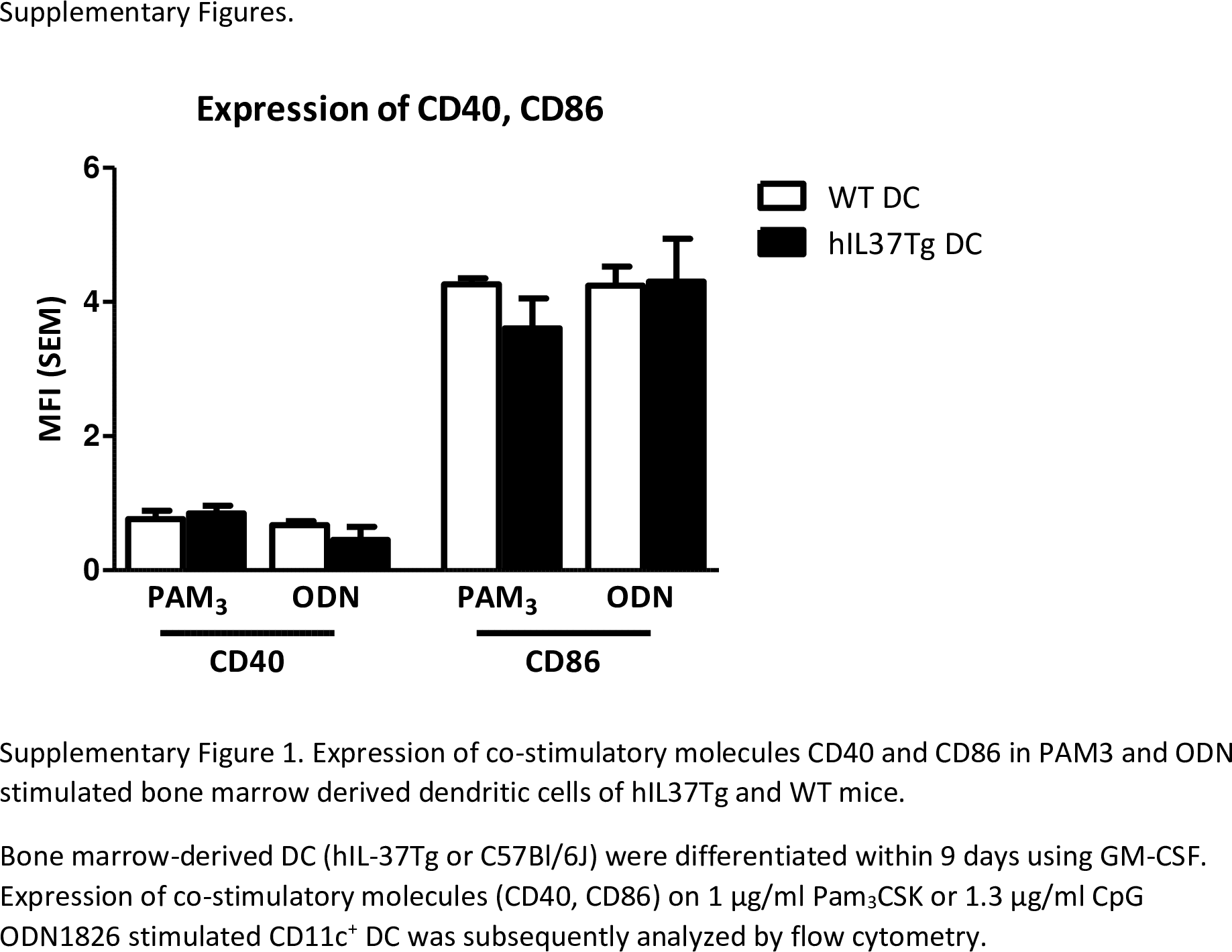

Supplement: S1 Fig — Bone marrow-derived DC (hIL-37Tg or C57BI/6J) were differentiated within 9 days using GM-CSF. Expression of co-stimulatory molecules (CD40, CD86) on 1 μg/ml Pam3CSK or 1.3 μg/ml CpG ODN1826 stimulated CD11c+ DC was subsequently analyzed by flow cytometry. (TIF) [file pone.0251809.s001.tif]
